# Obesity and Obesity-Related Comorbidities in a Canadian First Nation Population* *This article is part of a joint publication initiative between *Preventing Chronic Disease* and *Chronic Diseases in Canada*. *Preventing Chronic Disease* is the primary publisher, while *Chronic Diseases in Canada* is the secondary publisher.


**Published:** 2010-12-15

**Authors:** Sharon G. Bruce, Natalie D. Riediger, James M. Zacharias, T. Kue Young

**Affiliations:** University of Manitoba, Department of Community Health Sciences; University of Manitoba, Winnipeg, Manitoba, Canada; University of Manitoba, Winnipeg, Manitoba, Canada; University of Toronto, Toronto, Ontario, Canada

## Abstract

**Introduction:**

Rates of obesity are higher among Canada's Aboriginal First Nations populations than among non-First Nations populations. We studied obesity and obesity-related illness in a Manitoba First Nation community.

**Methods:**

We conducted a screening study of diabetes and diabetes complications in 2003, from which we drew a representative sample of Manitoba First Nations adults (N = 483). We assessed chronic disease and chronic disease risk factors.

**Results:**

Prevalence of obesity and associated comorbidities was higher among women than men. By using multivariate analysis, we found that factors significantly associated with obesity among women were diastolic blood pressure, insulin resistance, and employment status. Among men, factors were age, apolipoprotein A1 level, apolipoprotein B level, and insulin resistance. Seventy-five percent of study participants had at least 1 of the following conditions: obesity, dyslipidemia, hypertension, or diabetes. Comorbidity was high even among the youngest age groups; 22% of men and 43% of women aged 18 to 29 had 2 or more chronic conditions. Twenty-two percent of participants had undiagnosed hypertension. Participants with undiagnosed hypertension had significantly more chronic conditions and were more likely to have microalbuminuria than were those without hypertension. The number of chronic conditions was not significantly different for participants with newly diagnosed hypertension than for those with previously diagnosed hypertension.

**Conclusions:**

The prevalence of obesity and other chronic conditions in the study community is high, especially considering the number of young people. Community-based interventions are being undertaken to reduce the excessive rate of illness.

## Introduction

The Canadian First Nations population has poorer overall health than does the general Canadian population (1), specifically in terms of chronic diseases, chronic disease risk factors (2), and injuries and accidents (3). In Canada, First Nations peoples are 1 of 3 constitutionally recognized Aboriginal groups; the other 2 are the Métis and the Inuit. In this article, we use the term *Aboriginal* to report on research that included 2 or more of these distinct groups if no distinction was made between the groups in the analysis. However, if the research included only 1 group, we have identified that group. According to the 2005-2006 Canadian Community Health Survey, the prevalence of obesity among people who self-identified as Aboriginal and who did not live on reserve land was 20% in Canada's north (Yukon, Northwest Territories, and Nunavut) and 23% in the rest of Canada (4).

Obesity prevalence appears to be higher among First Nations people living on reserves. In Sandy Lake, Ontario, the prevalence of obesity (body mass index [BMI] ≥30 kg/m^2^) was 50% for men and 65% for women (5). Furthermore, in a First Nation community in Quebec, 91% of study participants from a sample of 172 were abdominally obese (6). The prevalence of obesity was 55% among a sample of Alberta First Nations people and 49% among a sample of Métis people (7).

Prevalence of obesity-related comorbidities is also high among Canadian First Nations peoples. The prevalence of diabetes among Canadian First Nations populations is 3 to 5 times higher than among the general Canadian population (5-8). Hypertension, dyslipidemia, metabolic syndrome, and diabetes complications such as cardiovascular disease (CVD), stroke, retinopathy, neuropathy, and nephropathy are also major contributors to poor health (2,5-10). CVD is one of the leading causes of death in Canada, and Aboriginal populations have twice the CVD death rate of non-Aboriginal populations (2). In a random sample, the rate of CVD was 18% among Canadian Aboriginal people and 8% among people of European ancestry (2).

Despite the evidence of excess obesity, diabetes, and related metabolic conditions among Canada's First Nations populations, few researchers have investigated their coexistence in this population. Our purpose was to explore the magnitude and effect of obesity and obesity-related comorbidities in a Manitoba First Nation.

## Methods

Our methods have been previously described (10). Briefly, 483 eligible residents of a Manitoba First Nation community volunteered in 2003 to participate in a screening study for diabetes and diabetes complications. A total of 1,356 eligible participants included nonpregnant adults aged 18 years or older who were Registered Indians and who were residents of the community. Our sample (36%, 483 of 1,356) is representative of eligible participants by age and sex (10). A registered nurse drew venous samples to measure glucose, hemoglobin A1c, insulin, total cholesterol, high-density lipoprotein (HDL) cholesterol, triglycerides, apolipoprotein A1 (apoA1), total apolipoprotein B (apoB), and homocysteine levels from fasting participants (low-density lipoprotein [LDL] cholesterol was calculated).

A registered nurse or trained research assistant administered a 17-item questionnaire that included standard demographic data (age, sex, employment status, education level), current and past smoking status, number of cigarettes smoked per day, previous diagnosis of diabetes and hypertension ("Have you ever been told by a doctor that you have diabetes? How long have you had diabetes?"), and current medication use. Standard techniques were used to obtain anthropometric measures (11). Height was measured via metric wall tape and set square to the nearest 0.5 cm; weight was measured on a balance scale to the nearest 0.1 kg; waist circumference was measured at noticeable waist narrowing or at the level of the 12th rib, to the nearest 0.5 cm; and hip circumference was measured at the level of the symphysis pubis and the largest area of the buttocks to the nearest 0.5 cm (11).

Abdominal obesity was defined as waist circumference greater than 102 cm for men and greater than 88 cm for women (10). Diabetes was defined as a fasting plasma glucose of 7.0 mmol/L or higher, or a previous diagnosis; impaired fasting glucose was defined as a fasting plasma glucose of 6.1 to 6.9 mmol/L (12). Hypertension was defined as systolic blood pressure higher than 140 mmHg or diastolic blood pressure higher than 90 mmHg, or a previous diagnosis. Dyslipidemia was defined as a plasma triglyceride level of 1.7 mmol/L or higher and HDL cholesterol level of 1.03 mmol/L or less for men or 1.30 mmol/L or less for women. Metabolic syndrome was defined using Adult Treatment Panel III criteria (13). Insulin resistance was estimated through the homeostasic model assessment (HOMA), which is calculated as follows: [(insulin [pmol] x 0.139) x (glucose [mmol/L]/22.5)]. Microalbuminuria was defined as an albumin-to-creatinine ratio higher than 2.0 mg/mmol for men and higher than 2.8 mg/mmol for women. Neuropathy was defined as presence of numbness, tingling, pain, and loss of protective sensation determined through application of the 10-g Semmes-Weinstein monofilament wire system (Sensory Testing Systems, Baton Rouge, Louisiana) (14). A registered nurse completed the foot examination and applied the 10-g monofilament. The University of Manitoba Health Research Ethics Board approved the project.

Statistical analyses were completed by using SPSS version 16 for Windows (IBM, Chicago, Illinois). We used χ^2^ tests to detect differences between the sexes for chronic disease prevalence, risk factors, and sociodemographic variables. We compared differences between the sexes on variables that were continuously distributed by using *t* tests or Mann-Whitney tests for variables with a nonnormal distribution. Differences in the number of chronic health conditions by age group and sex and number of comorbidities by hypertensive status were determined by using χ^2^ tests. Tests were 2-tailed and differences were considered significant at *P* < .05. We used logistic regression to estimate odds ratios (ORs) for obesity and microalbuminuria with 95% confidence intervals (CIs). Participants with missing values were excluded from analyses. No pattern was found for missing values by sex, age group, chronic disease, or risk factor variables.

## Results

The demographic and health status characteristics of the study sample describe a young population with low education and high unemployment (Table 1). The prevalence of smoking, diabetes, hypertension, and overweight and obesity was high among study participants. Waist circumference was available for 259 of the 264 obese participants; 96% (250 of 259) had waist circumferences that placed them at high risk for adverse health outcomes (9). We found no significant differences between men and women in prevalence of diabetes or hypertension. However, the prevalence of dyslipidemia among women (38%) was significantly higher than among men (26%).

### Overall obesity and abdominal obesity

**Figure 1:**
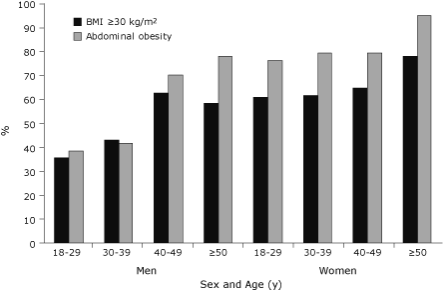
Prevalence of obesity by age and sex in a Canadian First Nation population. BMI, body mass index. Abdominal obesity was defined as waist circumference greater than 102 cm for men and greater than 88 cm for women.

We used BMI and waist circumference to classify participants as obese by age and sex (Figure 1). Almost 50% of men and 65% of women were obese as defined by BMI, and 53% of men and 81% of women had abdominal obesity. Obesity was more common among women than men according to BMI (χ^2^ = 14.62, *P* < .001) and abdominal obesity (χ^2^ = 41.38, *P* < .001). The prevalence of BMI ≥30 kg/m^2^ was higher among women aged 18 to 29 years than among men of the same age group (χ^2^ = 9.06, *P* < .01). Abdominal obesity was significantly more common for women than for men in all age groups except 40 to 49 years. Three-quarters of women aged 18 to 29 years had abdominal obesity (Figure 1).

**Table:** 

**Sex**	**Age, y**	**BMI ≥30 kg/m^2^, %**	**Abdominal Obesity, %**
Men	18-29	36	38
	30-39	43	41
	40-49	62	70
	≥50	58	78
Women	18-29	61	76
	30-39	61	79
	40-49	65	79
	≥50	78	95

Given the differences in obesity between men and women and the high prevalence of abdominal obesity, we determined factors associated with abdominal obesity for each sex by using multivariable backward stepwise logistic regression. Variables included in the models were those that were significantly associated with abdominal obesity in bivariate analyses. For women those variables were age; systolic and diastolic blood pressure; triglyceride, apoA1, and apoB levels; insulin resistance; education; and employment status. For men variables included in the model were age; systolic and diastolic blood pressure; triglyceride, apoA1, and apoB levels; insulin resistance; and microalbuminuria  (Table 2).

For women, the odds of abdominal obesity increased with diastolic blood pressure and insulin resistance. In addition, the odds of obesity were lower for women who were employed than for those who were unemployed. Among men, abdominal obesity was associated with increasing age, insulin resistance, lower apoA1, and higher apoB levels.

### Comorbidities

**Figure 2:**
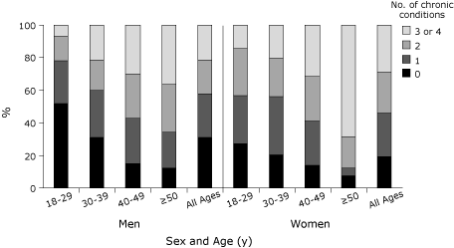
Percentage of sample with chronic conditions (obesity, diabetes, hypertension, dyslipidemia) by age and sex in a Canadian First Nation population. Because of rounding, percentages may not total 100.

We determined the extent of comorbidity among this population for 4 chronic conditions: obesity, diabetes, hypertension, and dyslipidemia. The distribution of chronic conditions by age and sex (Figure 2) showed that women aged 18 to 29 and aged 50 or older had significantly more chronic conditions than men of the same age groups. Twenty-two percent (16 of 73) of men and 43% (30 of 69) of women aged 18 to 29 had 2 or more preventable chronic conditions. Among participants with abdominal obesity, 48% (147 of 303) had hypertension and 35% (111 of 313) had diabetes. Thirty-seven percent (54 of 147) of the hypertension and 26% (29 of 111) of diabetes cases among these participants were undiagnosed.

**Table:** 

**Sex**	**Age, y**	**No. of Comorbidities, %**			
		**0, %**	**1, %**	**2, %**	**3 or 4, %**
Men	18-29	52	26	15	7
	30-39	31	29	18	21
	40-49	15	28	28	30
	≥50	12	22	30	37
	All ages	31	26	21	21
Women	18-29	27	29	29	14
	30-39	20	36	23	20
	40-49	14	27	27	31
	≥50	7	5	19	68
	All ages	19	27	25	29

### Undiagnosed hypertension

Overall, 22% (72 of 337) of study participants had undiagnosed hypertension. We compared the extent of comorbidity for participants with newly diagnosed hypertension and 2 groups: 1) participants who were not hypertensive and 2) participants with a previous diagnosis of hypertension (Table 3). Participants with newly diagnosed hypertension were significantly more likely to have more chronic conditions than were the normotensive participants. However, we found no significant differences in extent of comorbidity between those with newly diagnosed hypertension and those with previously diagnosed hypertension. In terms of outcomes, the adjusted odds of microalbuminuria among people with newly diagnosed hypertension were almost 2 times higher than among those without hypertension. The adjusted odds of microalbuminura among those with previously diagnosed hypertension were almost 5 times higher than among those without hypertension [<.001 in Table].

## Discussion

The prevalence of obesity in the study population is among the highest reported for a Canadian First Nations community on a reserve (6,7) and is substantially higher than that among the general Canadian (4) and off-reserve Aboriginal populations (4,15), whether the studies used self-reported data (4,6) or direct measurement (15). The high prevalence of obesity in the study population is concerning given the etiologic role of obesity in diabetes, heart disease, stroke, and some cancers. The prevalence of diabetes that we found is one of the highest reported among Canadian First Nation populations (6,7,16).

One finding of concern is the high prevalence of obesity among young adults, especially young women of reproductive age. The relationships between maternal obesity and gestational diabetes, type 2 diabetes, poor birth outcomes, and development of obesity and type 2 diabetes among offspring are well documented (17-20). Thus, the prevalence of obesity in this young study population warrants intervention. These findings are important for 2 reasons: 1) participants developed chronic conditions at young ages, and 2) hypertension and diabetes cases were undiagnosed among a large proportion of obese participants.

Results from logistic regression confirmed established associations between obesity and plasma lipid levels, hypertension, insulin resistance, and sociodemographic factors in the study population. The sex-specific regression analyses did not include lipids for abdominal obesity among women. We offer 2 possible reasons for this. First, the prevalence of abdominal obesity was high among women in all age groups but the presence of abnormal lipid levels was not. These age differences may have been blunted because our outcome (obesity) was present in all age groups. Second, previous research has shown significant sex differences in the relationship between adiposity and plasma lipids (21). Because abnormal lipid levels did occur among women, this finding warrants further examination.

We found a high prevalence of comorbidity even among the youngest age groups. The Diabetes and Related conditions in Urban Indigenous people in the Darwin region (DRUID) study also found high numbers of cardiovascular comorbidities among Australian Aborigines, and a higher number of comorbidities with increasing age (22). A large proportion of the study participants had undiagnosed diabetes and hypertension, despite the known strong correlations among obesity, diabetes, dyslipidemia, and hypertension (23) (we could not determine the extent of undiagnosed dyslipidemia among study participants because we did not ask them to self-report abnormal lipid levels). In a previous study, risk factors for not having blood pressure measured included male sex, never being married, not having a regular physician, being younger, and belonging to an Aboriginal or other ethnic minority group (24). In our study, the likelihood of not having hypertension diagnosed was higher for men (OR, 3.27; 95% CI, 1.74-6.10; *P* < .01) and younger participants (OR, 1.04; 95% CI, 1.01-1.07; *P* < .001).

In our study, the undiagnosed hypertension was not benign. The extent of comorbidity among participants with newly diagnosed hypertension was similar to that for those with previously diagnosed hypertension. In addition, the risk for microalbuminuria was significantly higher among participants with newly diagnosed hypertension compared with those without hypertension but not significantly different between those with newly diagnosed hypertension and those with previously diagnosed hypertension. This suggests that newly diagnosed hypertension among participants had existed for some time. The association between hypertension and outcomes such as CVD and stroke warrants vigilant screening on the part of health care providers, especially in high-risk populations. Some participants in our "newly diagnosed" group may have been told by a physician that they did have hypertension, but they may not have remembered or they may have not understood. However, none were receiving antihypertensive treatment, so they probably had not received a hypertension diagnosis before our study.

The study is subject to limitations. First, our sample was based on volunteers and therefore may not be representative of the community as a whole or of other Canadian First Nations communities. A screening study based on a volunteer sample may attract primarily healthy people who are motivated to learn more about their health, resulting in an underestimation of illness. On the other hand, a screening study can attract people who already have health problems and are seeking additional medical assistance, which may result in an overestimation of the prevalence of illness in a population. We do not think our sample was overrepresented by either group because men and women were equally represented, and the age distribution of our sample matched that of the eligible population (10). Another indication that the prevalence of illness in the community was not overstated is that only half of the community members known to have diabetes participated in the study. None of the 15 people with end-stage renal disease participated, and only 3 of 10 community members with amputations participated (10). The prevalence of chronic disease and risk factors that we report are not substantially out of line with previous research.

A second limitation is the use of a fasting glucose test rather than a glucose tolerance test. More people with diabetes may have been identified if 2-h glucose tolerance tests were conducted. However, our protocol is acceptable for epidemiologic research. A third limitation is that we did not validate the self-reported hypertension or diabetes status measures with local health care providers, so we may have underestimated self-reported prevalence and therefore overestimated undiagnosed cases. However, we have previously reported lack of adherence with standards of care in this community in relation to foot examinations among people with diabetes (10), so participants may not have been tested for diabetes and hypertension even when indicated. Finally, the study is cross-sectional, so we cannot infer the temporal sequence of events.

The prevalence of obesity in this population is among the highest reported among Canadian First Nation populations, particularly among women in their reproductive years. The extent of obesity-related comorbidity in this population is high even among young adults, and women at almost every age have a significantly higher rate of comorbidity than do men. A sizable proportion of participants have undiagnosed hypertension that may have been present for some time, given the significant associations with the other chronic diseases and microalbuminuria. The prevalence of cardiovascular and renal disease risk factors in this population may portend a larger prevalence of cardiovascular and renal disease. In addition, given the influence of maternal obesity and diabetes on the health of offspring, an increase in childhood obesity and type 2 diabetes could occur in the community.

An increasing prevalence of obesity and obesity-related conditions is not inevitable, however. Many prevention activities are under way. First, a research intervention in the community is focused on preventing gestational diabetes through controlling weight gain during pregnancy with exercise and diet. Second, the community operates a fitness center that has good equipment and instruction. Third, the health center offers education on diet, exercise, and wellness. Fourth, walking groups for youth and adults are organized through the health center. Fifth, activity programs for young people operate out of the local schools. However, given the well-established effect of obesity on health, continued surveillance of chronic disease and risk factors is warranted, as are further health promotion and health education initiatives. We continue to work with the community to develop and evaluate primary and secondary prevention activities.

## Figures and Tables

**Table 1:** Characteristics of First Nation Population (N = 483), Manitoba, Canada, 2003

Characteristic^a^	Value
**Sex, n (%)**	
Men	230 (48)
Women	253 (52)
**Age, y, mean (SD)**	37.8 (12.3)
**Education (n = 469), n (%)**	
Grade 9 or higher	220 (47)
Lower than grade 9	249 (53)
**Employment status (n = 476), n (%)**	
Employed	137 (29)
Unemployed	339 (71)
**Ever smoked (n = 477), n (%)**	
Yes	391 (82)
No	86 (18)
**Current smoker (n = 471), n (%)**	
Yes	349 (74)
No	122 (26)
**BMI, kg/m^2 ^(n = 468), n (%)**	
<25.0	76 (16)
25.0-29.9	128 (27)
≥30.0	264 (56)
**Metabolic syndrome^b ^(n = 475), n (%)**	252 (53)
**Abdominal obesity^c ^ (n = 464)**	313 (68)
**Diabetes^d ^(n = 483), n (%)**	140 (29)
**Hypertension^e ^(n = 472), n (%) **	201 (43)
**Dyslipidemia^f ^(n = 483), n (%)**	155 (32)
**Microalbuminuria^g ^(n = 466), n (%)**	94 (20)

Abbreviations: SD, standard deviation; BMI, body mass index.

a Numerators vary from 464 to 483 because not all participants completed the full protocol.

b Defined using Adult Treatment Panel III criteria (13).

c Defined as >102 cm for men and >88 cm for women.

d Defined as a previous diagnosis or fasting blood glucose ≥7.0 mmol/L.

e Defined as systolic blood pressure >140 mm Hg or diastolic blood pressure >90 mm Hg or previous diagnosis.

f Defined as plasma triglyceride level ≥1.7 mmol/L and HDL cholesterol level ≤1.03 mmol/L for men or ≤1.30 mmol/L for women.

g Defined as an albumin-to-creatinine ratio >2.0 mg/mmol for men and >2.8 mg/mmol for women.

**Table 2:** Odds of Abdominal Obesity by Sex, First Nation Population (N = 483), Manitoba, Canada, 2003

Sex	Risk Factor	β (SE)	OR (95% CI)	*P* Value^a^
Women	Currently employed	−1.16 (0.45)	0.31 (0.13-0.76)	.01
	Diastolic blood pressure	0.05 (0.02)	1.05 (1.01-1.10)	.03
	Insulin resistance	1.14 (0.205)	0.31 (0.13-0.76)	.01
Men	Age	0.05 (0.01)	1.05 (1.02-1.08)	.001
	ApoA1	−3.06 (1.20)	0.05 (0-0.49)	.01
	ApoB	1.54 (0.68)	4.64 (1.22-17.65)	.02
	Insulin resistance	0.33 (0.08)	1.40 (1.19-1.63)	<.001

Abbreviations: SE, standard error; OR, odds ratio; CI, confidence interval; apo, apolipoprotein.

a Calculated by logistic regression.

**Table 3:** Comorbidities and Risk for Microalbuminuria by Hypertension Status, First Nation Population, Manitoba, Canada, 2003

Participants' hypertension status (N = 453)^b^	No. of Participants (%)					Risk for Microalbuminuria^a^		

	No. of comorbidities				*P* Value^c^	β (SE)	Odds Ratio	*P* Value^c^

	0	1	2	3				
No hypertension (n = 263), n (%)	111 (42)	88 (33)	50 (19)	14 (5)	1 [Reference]	1 [Reference]	1.000	1 [Reference]
Newly diagnosed hypertension (n = 72), n (%)	17 (24)	20 (28)	19 (26)	16 (22)	<.001	0.653 (0.48-0.82)	1.921	<.001
Previously diagnosed hypertension (n = 118), n (%)	18 (15)	36 (31)	38 (32)	26 (22)	.510	1.542 (1.22-1.86)	4.673	<.001

Abbreviation: SE, standard error.

a Adjusted for age and sex. Those with newly diagnosed hypertension had no significant difference in risk for microalbuminuria compared with those with previously diagnosed hypertension.

b In this analysis, we included only participants for whom values were available for all variables.

c Calculated by using χ^2^ test.
